# A tabletop X-ray tomography instrument for nanometer-scale imaging: reconstructions

**DOI:** 10.1038/s41378-023-00510-6

**Published:** 2023-04-14

**Authors:** Zachary H. Levine, Bradley K. Alpert, Amber L. Dagel, Joseph W. Fowler, Edward S. Jimenez, Nathan Nakamura, Daniel S. Swetz, Paul Szypryt, Kyle R. Thompson, Joel N. Ullom

**Affiliations:** 1 National Institute of Standards and Technology, Gaithersburg, MD 20899 USA; 2 National Institute of Standards and Technology, Boulder, CO 80305 USA; 3 Sandia National Laboratories, Albuquerque, NM 87123 USA; 4 Department of Physics, University of Colorado, Boulder, CO 80309 USA

**Keywords:** Electronic devices, Physics

## Abstract

We show three-dimensional reconstructions of a region of an integrated circuit from a 130 nm copper process. The reconstructions employ x-ray computed tomography, measured with a new and innovative high-magnification x-ray microscope. The instrument uses a focused electron beam to generate x-rays in a 100 nm spot and energy-resolving x-ray detectors that minimize backgrounds and hold promise for the identification of materials within the sample. The x-ray generation target, a layer of platinum, is fabricated on the circuit wafer itself. A region of interest is imaged from a limited range of angles and without physically removing the region from the larger circuit. The reconstruction is consistent with the circuit’s design file.

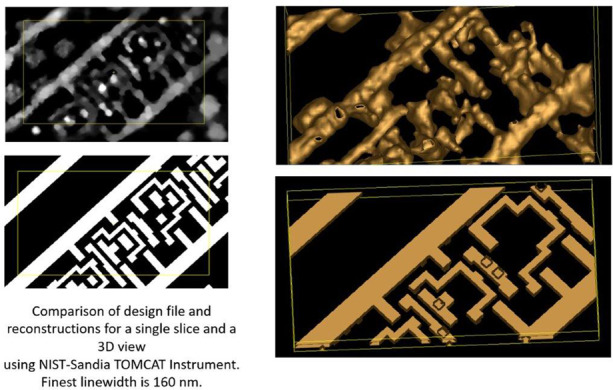

## Introduction

There are several reasons for the three-dimensional imaging of integrated circuit (IC) interconnects^1^. A manufacturer may be interested in process control, particularly in the case of new processes in the research and development phase. In a world in which the design and manufacture of chips are sometimes conducted by different organizations spanning the globe, there is a need to verify the design or detect counterfeit designs^2–4^. Reverse engineering is an additional motivation^1^.

Tomographic 3D imaging at the length scales required to analyze an IC is challenging for several reasons. Modern ICs contain a complex 3D matrix of features, with elements of wiring and transistors below 10 nm for some processes. X-ray sources of sufficient intensity must be confined to a spot not much larger than the desired resolution, and their position relative to an IC sample must be both maintained and measured with similar precision. Worse, the number of x-ray photons required to image a given volume of an optically thin sample scales as the inverse fourth power of the spatial resolution, while making an x-ray source ever smaller typically *decreases* the photon production rate by at least the inverse square of the resolution.

Because of the extreme intensity requirements, published 3D analysis of ICs at nanometer length scales has so far been dominated by research at synchrotron beamlines. Integrated circuit interconnects were initially imaged at synchrotrons using absorption contrast^5^ in the late 1990’s. Over the past two decades, the coherent nature of synchrotron radiation was exploited with the development of ptychography, a technique of stepping the beam over overlapping circular regions of the sample and allowing the radiation to propagate to the far field^6^. The last few years have seen application to integrated circuit interconnects with increasing sophistication^7–11^.

Despite the successes of synchrotron-based systems, there is also intense interest in laboratory instruments capable of nanotomography^12–16^. Integrated circuits have been imaged with electron microscopes, yielding first-depth information^17^, and later tomography^18^. A more recent development has been interleaving 2D electron microscopy with the application of a focused ion beam (FIB) to image circuits in three dimension by removing material in successive layers^19^. An SEM system with an electron target producing x-rays a few micrometers from a sample has also been used for tomographic reconstruction of integrated circuit interconnects, in combination with a FIB for validation and finer spatial resolution^15^. While promising, FIB-based approaches consume the sample during the measurement, which prevents follow-up analysis with other tools. A combined Scanning Transmission Electron Microscope (STEM) and Energy-Dispersive Spectrometer (EDS) has been used recently to create material-specific 3D reconstructions of a nanowire^20^. Laboratory systems are also being developed that combine x-ray tube sources with x-ray focusing optics. Commercial systems built around these technologies can readily provide image reconstructions with micrometer-scale resolution, with modern systems showing promise of achieving resolutions of several tens of nanometers over small sample volumes^21–23^.

Our approach to laboratory-scale nano-CT begins with the fabrication of a thin conversion material onto the IC sample itself, allowing the replacement of an x-ray source with a focused electron beam—in this case, a Scanning Electron Microscope (SEM). Superconducting cryogenic x-ray microcalorimeters are used for x-ray detection, specifically, the Transition-Edge Sensor (TES)^24^. The energy-resolving power of the TES reduces x-ray backgrounds and promises the ability to discriminate multiple chemical elements within a sample. Importantly, an IC sample is not consumed in the process, allowing for repeated CT measurements or for the introduction of other imaging or analytic tools.

Here, we present tomographic results from an integrated circuit interconnect using this hybrid electron and x-ray microscope^25–27^ and compare the reconstructed image to the Graphics Design System (GDS) file used to fabricate the IC. Our purpose in this work was to validate the combination of source, detector, sample preparation technologies and reconstruction algorithms as a complete tool. Although synchrotron-based instruments have made considerable achievements, laboratory-scale instruments enable broader access and are more compatible with the security requirements of governments and corporations.

## Results

**Fig. 1:**
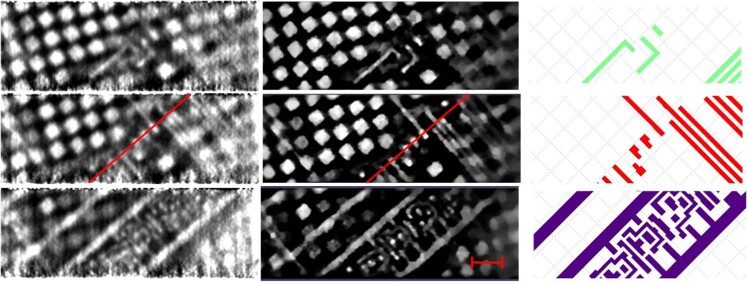
Reconstructions of metal layers M3 (top row), M2 (middle row), and M1 (bottom row) using (left) MLEM and summing the energy bands from 4.9 keV to 5.9 keV and 9.2 keV to 9.5 keV with 100 iterations and 1 pixel Gaussian blur, (middle) using the TomoScatt code and the 9.1 keV to 10.1 keV band for the selected slices, comparison with the original GDS design (right). According to the GDS, the large L in the top panel is 3.46 μm × 0.71 μm and the facing corner piece is 1.26 μm × 1.16 μm. Those lines have a width of 0.20 μm. The scale bar is 2 μm. The features that dominate the reconstructions but do not appear in the GDS file are CMP fill, which are not a part of the GDS design file as they are added by the foundry. The thin red lines running in the southwest-to-northeast direction across M2 reconstructions (middle row, first and second columns) are used for the plot in Fig. 2

Using the instrument described in Ref. ^27^ and techniques described in the Materials and Methods section below, we reconstructed images of an integrated circuit interconnect from a 130 nm Cu process^28,29^. This 130 nm process provides minimum feature sizes of 160 nm in the interconnect. Results for three slices are shown in Fig. 1 along with the design file. Reconstructions are given for the maximum likelihood-expectation maximization (MLEM) method^30^ and the Bayesian TomoScatt program^31^. Both show similar features, with TomoScatt showing better contrast and spatial resolution. While the reconstruction conditions are not identical, the results presented are the best available for each program.

All layers show a regular array of Chemical-Mechanical Polishing (CMP) fill interrupted by the circuitry. CMP fill in integrated circuits with copper wiring can be made out of copper or tungsten^32,33^. Its purpose is to provide a constant density of metal on each layer to reduce stresses through the circuit and to facilitate polishing. In the region near our circuit features of interest the CMP appear on a grid 1.3 μm × 1.3 μm at an angle of 24^∘^ degrees to the long wiring axis. They are approximately square, with a side of 0.7 μm. The grid is interrupted when any circuitry appears within 0.9 μm of wiring. The CMP fill is found in the reconstruction, but does not appear in the Graphics Design System (GDS) file.

The circuitry located in the center of the reconstruction region is also present in the design file. Some features are lost towards the edges of the images as expected for Region of Interest tomography, as discussed below in the subsection entitled “Laminar Sample”. The smallest circuit features, which appear in metal 1 (the bottom row of Fig. 1), between the two bus bars are also reconstructed successfully.

**Fig. 2:**
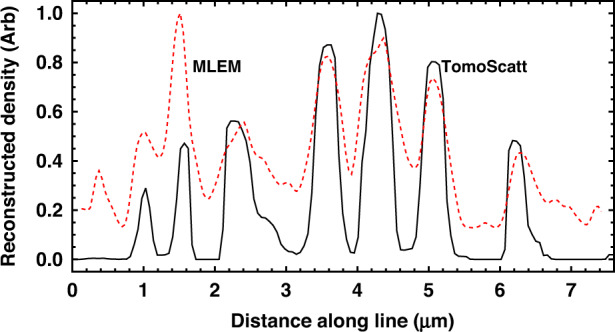
A 1D plot of reconstructed values along the red lines indicated in Fig. 1

Lineouts are shown in Fig. 2. (A “lineout” is a plot of values in an image or reconstruction along a specified line.) The contrast and resolution are seen to be somewhat better for the TomoScatt reconstructions than for the MLEM, although both can resolve the circuits. We will focus on the TomoScatt results in the rest of this article.

**Fig. 3:**
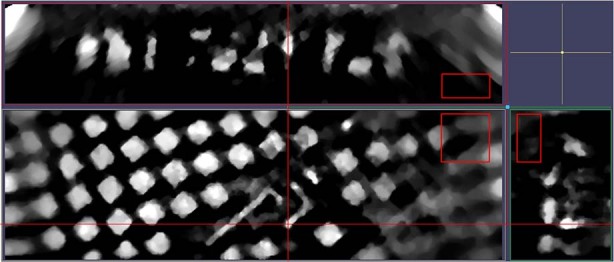
Orthogonal view of 9.1 keV to 10.1 keV reconstruction at top slice shown in Fig. 1. If the left bottom panel is taken to be in the *x*-*y* plane, then the panel at the right is in the *y*-*z* plane (i.e., needs to be rotated 90^∘^ about the *y* axis) and the top left panel is in the *x*-*z* plane (i.e., needs to be rotated 90^∘^ about the *x* axis). The red lines pass through the common point on all three panels. The three red boxes are each 2 μm on a side. The images are given in the voxel space, i.e, the *xz* and *yz* views are not equal-aspect-ratio views in length, but they are equal-aspect in voxels. Created with IMOD^52^

The top layer of Fig. 1 is reprinted in Fig. 3, without cropping. The blur at the ends is characteristic of the region of interest tomography^34^, as discussed below in Subsection “Laminar Sample”. The two orthogonal views show the depth into the material. The structures are shown in the voxel space, which has a 1:1:2 aspect ratio. Hence, distances in the *z* direction are twice what is shown. This affects the top panel and right panel, but not the main panel in the lower left of Fig. 3.

**Fig. 4:**
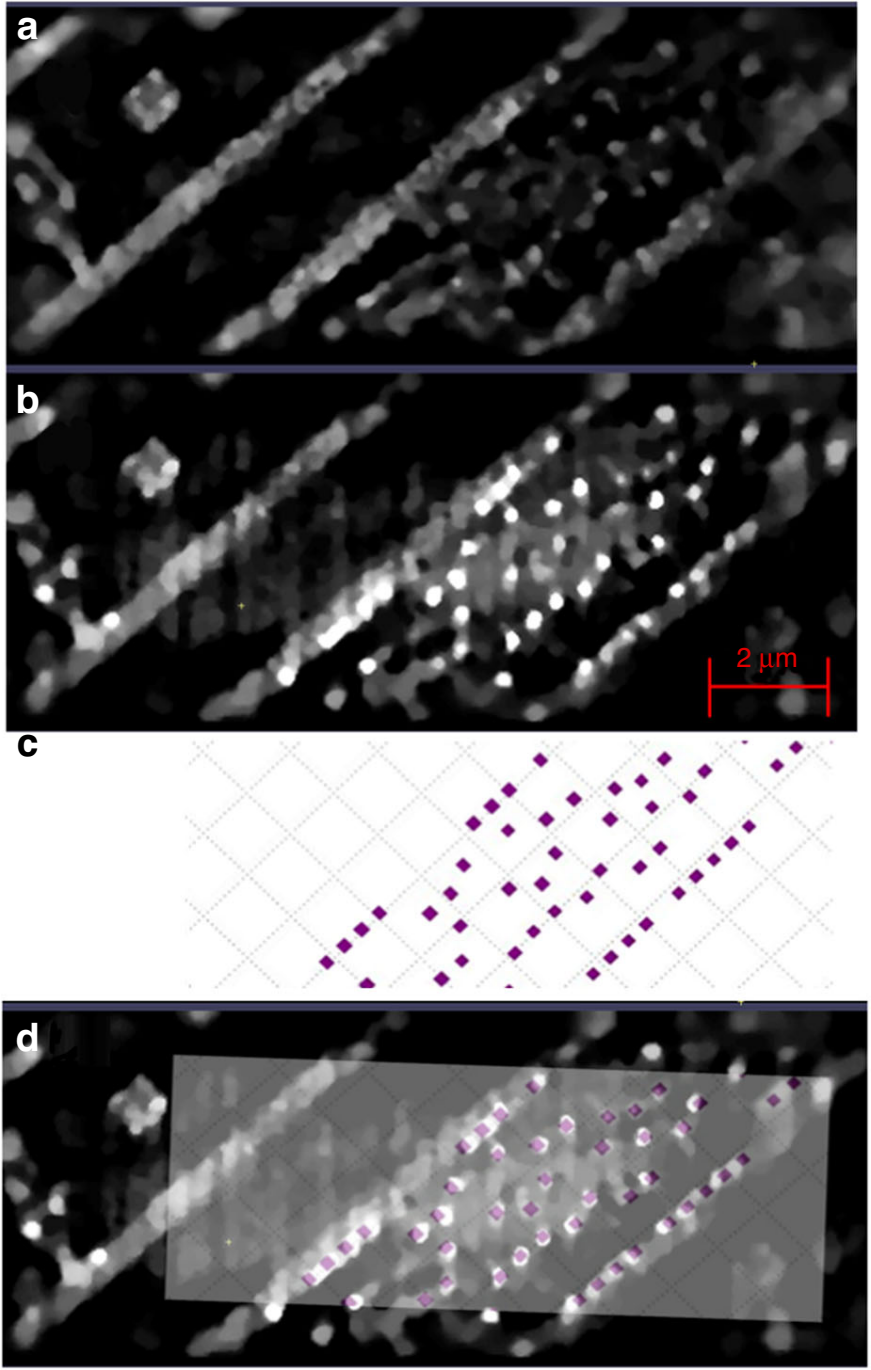
First via layer, which touches the device layer, as reconstructed (**a**) using 9.1 keV to 10.1 keV x-rays, (**b**) using 5.4 keV to 6.4 keV and 9.1 keV to 10.1 keV x-rays, (**c**) expected vias from GDS layer, and (**d**) expected vias from panel (**c**) shown overlaid on reconstructed layer of panel (**b**). The scale bar is 2 *μ*m

The via layer touching the active circuit elements is shown in Fig. 4. Using the reconstruction with the 9.1 keV to 10.1 keV band, as shown in Fig. 4a, the circuit from the first wiring layer has bled through and the vias are not prominent. However, by adding the 5.4 keV to 6.4 keV band, the vias become very prominent, as shown in Fig. 4b. Some of the vias are seen to be merged, which is an artifact of the reconstruction. The Bouman-Sauer^35^ prior penalizes reconstructions that oscillate from a high value to a low value. Since the physical structure in fact does this, it is an easy mistake for the code to make. We conclude that the vias are not made of copper, since the copper wires do not show an enhancement in contrast when the lower energy band is added. The vias from the design file are shown in Fig. 4c. These are overlaid onto the circuit in Fig. 4d, showing that the vias are correctly identified.

## Discussion

We have demonstrated the reconstruction of integrated circuit interconnects with a minimum feature size of 160 nm using a laboratory instrument which was envisioned several years ago^25^ and has only recently come to fruition^27^.

Recent studies of the reconstruction of integrated circuits both at synchrotons^8^ and with laboratory sources^14^ obtain structures, which are argued to be reasonable based on general expectations, e.g., repeated memory structures. Here, we have structure-by-structure verification of the reconstruction with the design files. We are not aware of a similar comparison in the literature.

Beyond the hardware challenges, we needed to choose our tomography algorithms to be compatible with the limited angles delivered by the instrument. We selected two iterative algorithms, one, MLEM, which was more robust and a second, TomoScatt,^31^ which more closely models the physics. The Bayesian TomoScatt code ultimately produced a reconstruction with higher contrast and better spatial resolution compared to the MLEM code. However, the MLEM was more robust in the sense of being less sensitive to the exact assumptions (e.g., the normalization) than TomoScatt. The MLEM code therefore proved to be invaluable in the earlier stages of the project when we did not have fully refined estimates of all relevant operating conditions.

TomoScatt uses a Bayesian prior suited to the material inspection problem^36^. Although the minimization of total variation^37^ is more widely used now, the work of Sauer and Bouman is more compatible with gradient-based optimization methods and, if the constants are chosen to mimic the derivative, gives a nearly identical penalty function.

Ideally in tomography, the sample is physically isolated. Here, the region of interest is connected to the rest of the integrated circuit interconnect. We followed the practice of having an inner scan area with more dense sampling than an outer scan area^38^. We also mounted the sample near 45^∘^ so that the wiring in the *x* and *y* directions would be equally well resolved^39,40^. In practice, an angle of 41^∘^ between the long wiring direction and the long scan direction was observed.

By counting the number of observations, we could choose a reasonable voxel size. We found having up to a factor of two more degrees of freedom than observations is a good operating point. We chose a 1:1:2 aspect ratio for the voxels because (a) we have a viewing angle of about 90^∘^, we expect elongation in the *z* direction, and (b) we expected the sample itself to be elongated by up to 2:1 in the same direction^28^, so it is natural to have the voxel similarly elongated.

An energy-resolving detector was a key feature in our analysis. We originally intended to use only the Pt L_*α*_ line and were pleasantly surprised to learn that photons with nearby energies yield essentially identical reconstructions. This renders background subtraction unnecessary and opens the door to using larger energy bands. The band of 9.1 keV to 10.1 keV was chosen to include the Pt L_*α*_ line and to be above the Cu K edge. As a bonus, the attenuation of copper and tungsten are nearly identical in this range, so we could expect similar results regardless of which of these two popular materials the CMP fill^33^ was made from. While a TES array, which can resolve 20 eV, was envisioned early in the project^25^, retrospectively we see that resolution can be relaxed considerably. More conventional photon-counting detectors with a resolution of 100 eV in the 5 keV to 10 keV range^41^ may offer more choices in future instruments.

## Conclusions

We have successfully completed an x-ray CT reconstruction of 100 *μ*m^2^ from an integrated circuit with 160 nm copper features. The reconstruction is consistent with the known design of the circuit. The measurement used a novel combination of a commercial SEM, a nano-positioning stage, an IC sample prepared with an integrated electron-to-photon conversion layer, and an array of cryogenic microcalorimeters as an x-ray camera with high energy resolution. This work demonstrated use of an electron beam onto an integrated platinum target adhered to the IC wafer. The integrated target layer provides a magnification factor that is at once extremely stable and well-matched to the available sizes of detector pixel. The tomography was performed in the challenging regimes of low photon counts and with a range of angles limited to about ± 45^∘^. The benefits of the excellent energy resolution of TES detectors have not yet been employed to full advantage, but their wide spectral coverage can help discriminate the elements in future samples beyond the preliminary material identification shown here. Their excellent resolving power will be used in future measurements in which the two-dimensional target layer will be replaced by structured nanoscale targets composed of three or more distinct elements. The TES can readily distinguish the elements’ characteristic fluorescence lines, offering a form of multiplex advantage.

Although synchrotron-based CT instruments are likely to maintain substantial advantages over laboratory instruments in measurement speed and spatial resolution, they will inevitably remain a scarce resource. Some industrial and government laboratories may require an in-house capability for analyzing the 3D structure of IC samples. High-voltage electron microscopy has already proven itself in this regard^18^. The system described here^27^ has sufficient energy and spatial resolution to make it a promising complementary instrument.

## Materials and methods

### Experiment

**Fig. 5:**
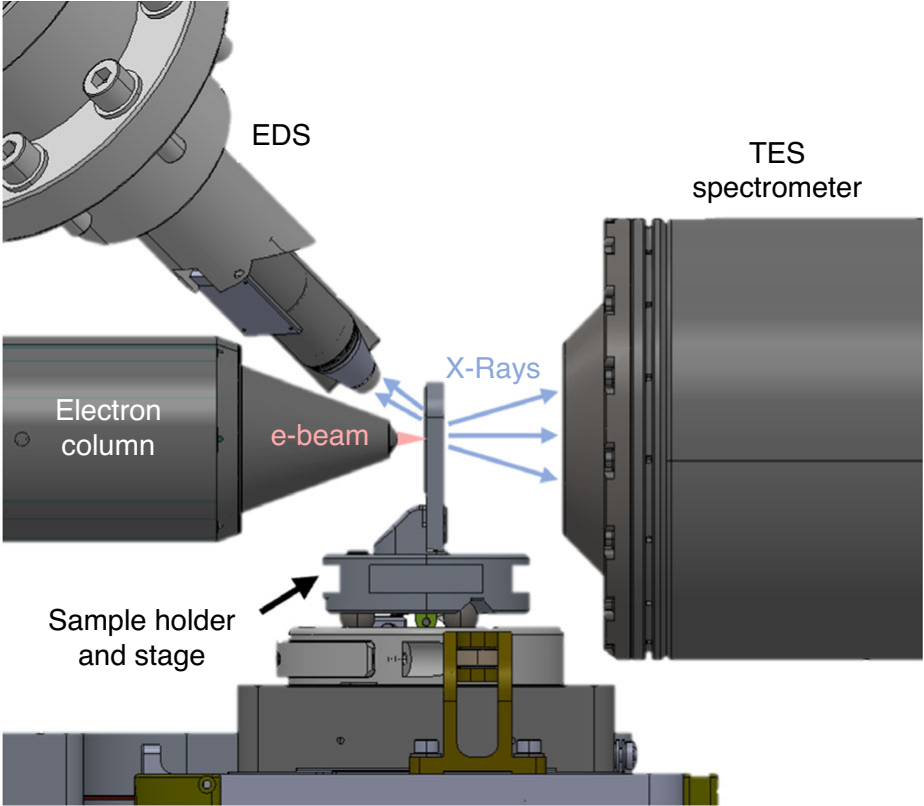
Sketch of experiment, including the electron beam (e-beam), Energy Dispersive Spectrometer (EDS), and TES Spectrometer. Figure reprinted from Ref. ^25^ with permission (creativecommons.org/licenses/by/4.0/)

**Fig. 6:**
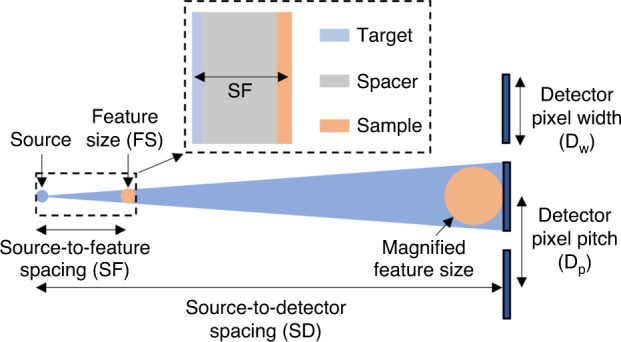
Close-up sketch of experiment, showing the source, a typical feature on the sample, and its magnified image on the detector. The detector pixel width *D*_*w*_ and pitch *D*_*p*_ are shown. The inset shows the Pt target layer, the silicon spacer layer, and the sample, an IC

Because the experimental instrumentation will be presented elsewhere^27^, we summarize the key features here for convenience. Earlier work includes predictive^25^ reports on the current instrument, but the current results represent the first images from the fully realized tool. A sketch of the experimental setup is given in Fig. 5 with a close-up in Fig. 6. Electrons at 25 keV from an electron microscope column are incident on the Pt target layer of the sample. X-rays are generated primarily in the target layer, which confines the majority of the generated x-rays to the nanoscale spot size of the electron beam. A small amount of additional x-rays are produced from a dispersed spot throughout the rest of the sample. A spacer layer of nominally 8.6 μm of silicon is left between the Pt target and the IC sample, which contributes to the final magnification of the system during imaging. At each projection angle, the stage is stepped to bring the electron beam across a series of inner and outer 2D regions to perform region-of-interest tomography. X-rays passing through the sample layers are collected using the TES spectrometer, which consists of approximately 200 functioning TES pixels. Each TES pixel operates as a single-photon counting detector with high resolving power, allowing the energy of each incident photon to be determined with high precision^24^. Fluctuations in the x-ray source term are monitored using the EDS detector, which collected back-going x-rays (i.e., photons not transmitted through the sample). This signal is proportional to the electron beam current, which varies by about 10%, enough to require continuous monitoring. This combination of nanoscale x-ray spot produced by the electron beam-target interaction, high magnification due to system geometry, and high resolving power single-photon detection by the TES spectrometer enables the nanoscale spatial resolution observed in the current tomographic reconstructions.

Prior to tomographic reconstruction, each TES detector is calibrated to allow conversion of individual x-ray pulses into an energy-calibrated x-ray spectrum. Details regarding the steps of TES calibration and processing can be found in prior publications^42,43^. Although there are approximately 220 working TES detectors, in practice, some give unreliable signals and are removed from consideration. During TES data processing, a detector is omitted from subsequent analysis if more than 20% of detected x-ray pulses in a single scan of the sample have anomalous pulse shape; this step identifies a small fraction of detectors which were not properly configured for high-resolution operation. Detectors for which most pulses are valid are then screened according to the detected Pt L_*α*_ count rate and the energy resolution at the Pt L_*α*_ emission line as determined by a line fit. If the x-ray count rate of a given detector is 25% higher or lower than the median across all detectors, or the energy resolution is not better than 60 eV, the detector is omitted from subsequent analysis. The TES spectrometer yields approximately 200 usable simultaneous spectra with a typical dwell time of 60 s before moving the stage. Additional details on TES data processing, data quality checks, and scan setup specific to this experiment can be found in Ref. ^27^.

The current instrumentation is limited to a single set of inner and outer region scans in a day due to settling of stage positional drift after angular movements and limitations on continuous TES spectrometer operation due constraints on cryostat operation. Additionally, certain scans were subject to abnormally high drift rates and recollected to improve data quality. In total, the data used in the current imaging demonstration was collected over 20 days. However, improvements to the TES cryogenics, TES spectrometer, and instrument hardware can drastically improve upon this imaging time. A TES instrument with 4 times as many active pixels as the current spectrometer has already been developed in an upgraded cryogenic refrigerator for this instrument,^44^ and another spectrometer with 12 times as many active pixels is underway^26^. Additionally, upgrades to the electron beam column and stage can improve positional stability and achievable count rates in future iterations of this tool. A more detailed discussion on the current limitations to imaging speed, as well as potential hardware upgrades to improve imaging speed and instrument capabilities can be found in Ref. ^27^.

### Justification of approximations

Our codes use projection tomography. Here, we consider both scatter corrections and diffraction.

Scatter corrections are of considerable importance in medical tomography, but they have little importance here. (We compare to medical tomography since it is the original and still most common application of tomography.) Because we have relatively high *Z* (i.e., high atomic number) materials, the scattering is less important. Moreover, when dealing with low energy x-rays (e.g., near 10 keV), photoabsorption dominates the cross section over scattering. Between the two effects, scattering is negligible.

Fresnel diffraction occurs when the Fresnel number is below 1. Otherwise, geometric optics applies. The Fresnel number is defined as *F* = *a*^2^/(*λz*), where *a* is the smallest feature size, *λ* is the x-ray wavelength, and *z* is the propagation distance. In the case of a high magnification system, the Fourier magnification theorem^45^ tells us that the relevant propagation distance is from the source to the sample. The much larger distance from the sample to the detector is irrelevant. In the experiment, *λ* = 0.124 nm at 10 keV, *z* ≈ 10 *μ*m, so geometric optics applies for *a* > 35 nm. The smallest wire size in our sample is 160 nm. Hence, the system is in the regime of geometric optics, although not by a great margin. Looking ahead, if an integrated circuit with finer feature sizes were imaged, the Fresnel number could easily be substantially less than 1, leading to significant diffraction effects^46^.

#### Motion

Here, we give an analytic estimate of the allowed uncertainty in the source position and compare that to data from the experiment. Consider an 1D pattern of material given by $f(x)={\cos }^{2}(kx)$ where *x* is a position and *k* is a wave vector. By definition, the Michelson visibility is given by

1 $$\nu =\frac{{f}_{\max }-{f}_{\min }}{{f}_{\max }+{f}_{\min }}$$

where the maximum and minimum of *f* are given by ${f}_{\max }$ and ${f}_{\min }$. If *f*(*x*) is convolved with a positional uncertainty given by a Gaussian distribution

2 $$p(\delta )=\frac{1}{\sqrt{2\pi }\sigma }{e}^{-\frac{{\delta }^{2}}{2{\sigma }^{2}}}$$

where *δ* is a deviation in position, *p* is a probability density function, and *σ* is its standard deviation, the resulting convolution is

3 $$F(x)=\frac{1}{2}+\frac{1}{2}{e}^{-2{k}^{2}{\sigma }^{2}}\cos (2kx).$$

For *F*, its fringe visibility is computed from Eqs. (1) and (3), and is given by

4 $${\nu }_{F}={e}^{-2{k}^{2}{\sigma }^{2}}.$$

If we require that the fringe visibility be at least $\frac{1}{2}$, the condition is

5 $$\sigma \le {\left(\frac{\ln 2}{2}\right)}^{1/2}\frac{1}{k}=\frac{{\left(2\ln 2\right)}^{1/2}}{\pi }a\approx \frac{3}{8}a$$

where $a=\frac{\pi }{2k}$ is the half-pitch of *f* (i.e., *a* is the feature size). Thus, if the standard deviation of the motion exceeds $\frac{3}{8}$ of the half-pitch, then there will be a significant loss of visibility. Although the choice of $\frac{1}{2}$ for the visibility threshold is arbitrary, since Eq. (4) has a Gaussian dependence on *σ*, relaxing the threshold will have little practical effect.

**Fig. 7:**
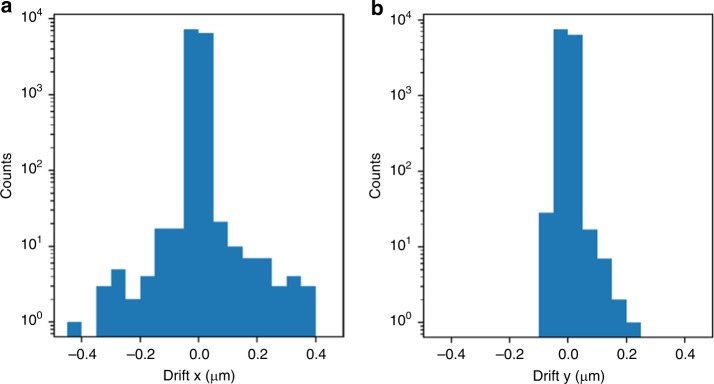
Histogram of drifts in (**a**) *x* and (**b**) *y* recorded in the experiment^27^. The means(standard deviations) are − 1(17) nm and − 1(13) nm for the two distributions, respectively

Next, we present the experimental drift obtained by measuring the position at the end of a dwell with the position at the beginning of the same dwell^27^ in Fig. 7. The combined standard deviation of 21 nm is only 13% of the minimum feature size of 160 nm expected in our sample, it is well below the suggested analytic upper bound for acceptable drift. This optimistic conclusion must be tempered by the fact that the distributions have long tails, i.e., are not strictly Gaussians, and some observations take place at least 160 nm from the nominal location. On the other hand, in practice, we minimize the effect of the drift by assigning the midpoint of the position at the beginning and the end of a dwell to represent the position of at particular measurement so we are less sensitive to the motion drift by perhaps a factor of 2. Simulation results suggest that the motion is not a significant issue in this analysis.

### Algorithms

Two codes with different algorithms are used in this work to make the results presented here more reliable. We discuss TomoScatt here and MLEM in the Appendix. Both MLEM and TomoScatt were implemented as a list-driven reconstruction algorithms where the input is list of observations, each containing the source position, detector pixel position, and detector pixel value. In the experiment, the source positions were programmed to be a set of rectangular arrays, but due to (monitored) drift the source positions are only approximately in such arrays. Similarly, the TES array is not strictly a simple rectilinear layout, particularly after unreliable detectors are omitted from consideration.

The code TomoScatt has been presented elsewhere^31^, so will be summarized here. The code implements the objective function of Sauer and Bouman^36^. The objective function includes a term that evaluates the log-likelihood that a given reconstruction is the optimal one by comparing projections with measurements. It also includes a second term representing a Bayesian prior that favors reconstructions with less oscillation, while allowing it to change quickly from one value to another. Using the value *p* = 1.1, which is recommended for the material inspection problem, and adjusting the weights so that the differences between neighboring voxels form an approximation to the absolute value of the gradient^46^, the resultant penalty function closely resembles the one used in the method of the minimization of total variation. A comparison of these methods in the context of medical tomography was presented earlier^47^.

Here, we did not use the scattering feature except briefly to confirm that scattering had a negligible effect on the reconstruction. For the present project, the code was modified from the version used in Ref. ^31^ to be table-driven, i.e., lists of source and detector positions are input. As discussed below, the source positions are taken to be the sum of the point position supplied by the experiment plus a set of offsets to describe the finite size of the source.

**Fig. 8:**
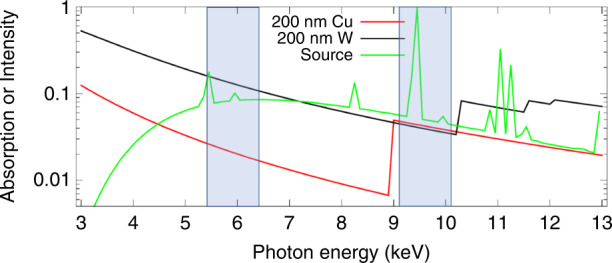
Spectrum as simulated with PENELOPE^50^ (green), normalized to 1 at its peak. Also shown are the absorption by 200 nm of copper (red) and tungsten (black)^53^. The transparent blue bands show the 5.4 keV to 6.4 keV and 9.1 keV to 10.1 keV bands used by TomoScatt in this study. In the event that color is not available, the source spectrum has distinct peaks and the tungsten absorption is generally larger than the copper absorption except near the marked band from 9.1 keV to 10.1 keV

The code was adapted to allow a treatment of a finite source by having several projections go from various source points to the center of a detector pixel. The intensity of each projection is added before comparison is made to experiment. The system matrix is written as a sum of projections indexed by ι, specifically

6 $${A}_{r\psi }=\mathop{\sum}\limits_{\iota }{c}_{\iota }{A}_{r\psi }^{(\iota )}$$

with ∑_ι_*c*_ι_ = 1 being a set of weights, here based on the source strength. The estimate of the experimental value is a generization of those given in Refs. ^31^ and^46^ to

7 $${I}_{j\psi }=\mathop{\sum}\limits_{\iota }{c}_{\iota }\int\,{{{\rm{d}}}}E\,D(E){I}_{j}^{(0)}(E)\exp \left(-\mathop{\sum}\limits_{ri}{f}_{ri}{\alpha }_{i}(E){A}_{r\psi }^{(\iota )}\right)$$

where *j* indexes the spectra, *ψ* indexes the observation conditions, *E* is the photon energy, *D* is the detector efficiency, ${I}_{j}^{(0)}$ is the incident flux at photon energy *E*, *i* indexes the basis materials in each voxel, $r$ indexes the voxels, *f* is the real number representing the amount of each basis material in a given voxel, *α* is a linear attenuation coefficient, and *A*^(ι)^ is the system matrix for a given projection. Eq. (7) reduces to its equivalent in the references if the set of *c*_ι_ only has a single value, *c*_1_. Only 1 material is used in the present work, but we have 1 or 2 spectra, represented by the blue bands in Fig. 8.

#### Role of GDS file

The design file was used only for comparison to completed reconstructions. It was not involved in the reconstruction process.

### Incident intensity

While attaching the target directly to the sample allows for high magnification^25^, the attachment implies that it is not possible to remove the sample from the beam to measure the incident flux, as is common in many tomography instruments. Nevertheless, TomoScatt requires the incident flux as a mean number of incident x-rays which would arrive at the detector in the absence of the sample. The MLEM program is more robust and can operate with unnormalized signals.

We break the problem into two parts: finding the average absolute flux and a relative, time-dependent flux. To obtain the absolute flux, we average the detected signal at normal incidence over all detectors and scan positions. We rely on the fact that the sample is optically thin to simplify the averaging process. We assume the sample is composed principally of copper and silica and estimate their relative abundance by comparing the total number of counts just above and just below the energy of the copper K edge (8.98 keV). Using this information and the attenuation coefficients from XCOM^48^, we find the average mass thickness of copper in the circuit to be 500 nm. This mean thickness is also consistent with both the observed angular dependence of the edge drop and the ratio of minimum to maximum transmitted x-ray intensities observed in two-dimensional radiographs (0.73, corresponding to 1.4 *μ*m of copper). The total EDS signal (summed over all energies) is proportional to the number of electrons striking the target, and is used to monitor any time variations in the relative flux. We refer the flux to the exit of the silicon spacer layer, so the spacer layer does not appear in the reconstruction.

The sample is somewhat thinner optically than the theoretical ideal transmission^49^ of *e*^−2^ ≈ 0.135. However, that analysis was based on a synchrotron scenario and assumed that the photon flux would be equal for all photon energies. Here, we cannot so easily vary the photon energy due to the copper K edge, the presence of the Pt L_*α*_ line, and the silicon spacer layer which acts as a significant filter for x-rays below 5 keV.

We use the PENELOPE^50^ simulation to give the spectral distribution after the spacer layer at normal incidence. We correct this to account for the additional attenuation in the spacer layer at other angles of incidence assuming the spacer layer is made of silicon with a thickness of 8.6 μm.

### Geometry

The reconstruction volume is taken to be 20 μm × 6 μm × 8 μm, with a voxel size of 40 nm × 40 nm × 80 nm, with the values ordered as *x*, *y*, and *z*. The number of voxels is 500 × 150 × 100 yielding a total of 7.5 million. In comparison, there are just over 2.631 million observations, i.e., projections, for the 9.1 keV to 10.1 keV band. (The number of observations is reduced by nearly one thousand if the 5.4 keV to 6.4 keV band is included, due to a very few additional exclusions of observations in that band. The code requires all bands to be observed.) Since there are more degrees of freedom (one per voxel) than constraints (observations), the codes rely on regularization. After normalization, there were 244(48) counts per observation, so the signal-to-noise per observation is 15.6. Similarly, there are 85.5 photons per voxel in this experiment. In the case of MLEM, the number of iterations is an implicit regularization. The contrast may be expressed as absorption of the in-band x-rays through 80 nm of copper vs. 80 nm of silica. The ratio is 0.982 to 0.993.

The center of the source layer is 10.31 *μ*m in *z* from the center of the reconstruction region. The center of the detector is 71 mm from the center of the reconstruction region. There is thus a nominal magnification of 6900 at normal incidence. Given that the TES detector pixels are 320 *μ*m × 305 *μ*m, the demagnified detector element is 46 nm × 44 nm at normal incidence, which also supports the choice of voxel size. After reconstruction, we found that the center of the sample appears about 4$\frac{1}{2}$ slices or 0.36 *μ*m closer to the source than the nominal value used. By adjusting the source-to-center distance in the program input, it is possible to move the reconstruction within the volume as expected.

Observations are taken every 7.5^∘^ from a nominal − 37. 5^∘^ to 45^∘^. The 7.5^∘^ spacing was chosen to match the angle subtended by the detector^27^. Based on the variation of the total number of counts, the nominal 0^∘^ is found to be at − 1.32^∘^ when adjusting fluxes for the sample thickness.

#### Laminar Sample

Ideally in tomography, the sample is in a finite region and all directions are accessible. In practice, our sample is laminar. We make observations over approximately ± 45^∘^, i.e., one-quarter of the ideal 360^∘^ viewing range, and the sample extends for a large distance outside of our viewing range. Consequently, some parts of the sample are only observed from a limited range of angles. Intuitively, we expect the center of the reconstruction region to be well described and the edges more poorly described.

**Fig. 9:**
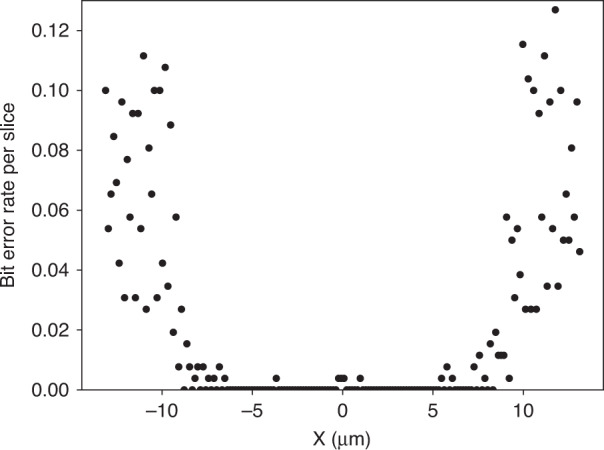
Error as a function of position in simulation at constant slice value

A test was made using a sample created with the CircuitFaker model^51^, a binary model with reconstructions from TomoScatt. The calculation used a 26.4 μm × 3.3 μm × 3.9 μm sample region with voxels of 150 nm × 330 nm × 150 nm with 13 angles sampled over ± 45^∘^. The detector was taken to be a 278 × 48 array of 575 *μ*m square pixels, with a magnification of 6888. These parameters form a preliminary model of our final experimental conditions. The results are shown in Fig. 9. The errors are almost entirely found near the edges of the sample in a layer approximately equal to the thickness of the sample. The central region is nearly error free. The distortions at the edges of Fig. 3 are in general agreement with the model results. (In most cases, we clip the edges before presenting the results.)

Chityala et al.^38^ addressed this problem by scanning in three nested rectangles with the relative doses in a ratio of 256:16:1. We use a similar strategy, although we only have two nested rectangles and use a dose ratio of 4:1.

#### Finite Source

PENELOPE^50^ simulations indicated that the median radial deviation of the source from its center in 2 dimensions is 115 nm, with significant tails extending to nearly 1 μm. Given that the minimum feature size is 160 nm, in order to obtain the best image, the finite source size needed to be taken into account. However, as the time to reconstruct the sample becomes dominated by calculating projections through numerous source points with linear scaling, it is important to minimize the number of sampling points.

These points were chosen as follows: the source distribution was taken to be a product of a 2D radial function with a uniform distribution in the azimuthal angle times a quadratic function in the 100 nm thickness of the Pt target layer. The quadratic function was found by evaluating the density of the source in five 20 nm layers and fitting. There is a variation of about ± 10% in intensity. The 2D radius of the points were chosen to be uniformly spaced in the cumulative density function of the radial distribution. For each point, the azimuth was taken to be an integer multiple of the golden angle, $n\pi (3-\sqrt{5})$ rad ≈ 138^∘^. The points in *z* were taken to be $n\sqrt{3}$ mod 1 × 100 nm. The number of samples used was 200. The choice of points in azimuth and *z* are quasi-random numbers (i.e., low-discrepancy sequences). We artificially considered the inner 25, 50, and 100 points, which have much smaller spreads. The reconstruction quality increases with the number of points, suggesting taking into account tails of the x-ray spot is an important consideration.

## Appendix: MLEM algorithm

Where no a priori knowledge of the object is known, an important iterative technique is the Maximum-Likelihood Expectation-Maximization (MLEM) algorithm. The algorithm is derived by alternating expectation and maximization steps and maximizes the likelihood for a Poisson data model^30^. The term “alternating” refers to the expression in the denominator of Eq. (8) and the equation is expressed as a composition of the expectation and maximization steps. The denominator is the forward projection step of the current solution. As a multiplicative algorithm, an iteration is computed by summing over all projections *ψ*

8 $${\hat{\theta }}_{r}^{(k+1)}={\hat{\theta }}_{r}^{(k)}\frac{1}{{s}_{r}}\mathop{\sum}\limits_{\psi }\frac{{g}_{\psi }}{{\sum }_{r}{\theta }_{r}^{(k)}{A}_{r\psi }}{A}_{r\psi }$$

where *r* is a voxel index, *θ*^(*k*)^ is the reconstruction at iteration *k*, *s*_*r*_ > 0 is the point sensitivity response of the imaging system, *k* is the iteration number, *g*_*ψ*_ is one of the projections through the sample (i.e., one measurement), and *A*_*r**ψ*_ is the system matrix. The system matrix is the geometric factor in the contribution of voxel *r* to projection *ψ*. A multiplicative model, and specifically MLEM, was chosen for a variety of reasons. No a priori knowledge of the imaged object is needed, thus avoiding extra unnecessary assumptions of the system. MLEM has been applied in several fields for its simplicity and robustness on sparse and noisy data. Even in the absence of Poisson distributed data, MLEM has a high probability of being able to reconstruct an object, albeit likely with artifacts or noise. Since it is multiplicative, if any regions outside the support are known, one can set those regions to zeros and will remain zero throughout the entire computation. (However, this knowledge is not needed to complete a reconstruction.) The only knowledge needed to execute MLEM for tomographic reconstruction is the imaging system geometry, including source positions, the detector pixel position, and the angles at which data are collected.

## References

[1] Botero UJ (2021). Hardware trust and assurance through reverse engineering: A tutorial and outlook from image analysis and machine learning perspectives. ACM J. Emerg. Technol. Comput. Syst..

[2] Mahmood K, Carmona PL, Shahbazmohamadi S, Pla F, Javidi B (2015). Real-time automated counterfeit integrated circuit detection using x-ray microscopy. Appl. Opt..

[3] Wilson R, Lu H, Zhu M, Forte D, Woodard DL (2021). Refics: Assimilating data-driven paradigms into reverse engineering and hardware assurance on integrated circuits. IEEE Access.

[4] Favata J, Shojaee SA, Shahbazmohamadi S (2020). 3D finite element simulation from non-destructive x-ray tomography and verification with novel mechanical testing and digital image correlation in-situ of focused beam microscope. Microsc. Microanal..

[5] Levine ZH, Kalukin AR, Frigo SP, McNulty I, Kuhn M (1999). Tomographic reconstruction of an integrated circuit interconnect. Appl. Phys. Lett..

[6] Pfeiffer F (2018). X-ray ptychography. Nat. Photo..

[7] Xi, X. et al. Tomographic observation of integrated circuit based on x-ray microscopy. In *AOPC 2015: Advanced Display Technology; and Micro/Nano Optical Imaging Technologies and Applications*, vol. 9672, 96720R (International Society for Optics and Photonics, 2015).

[8] Holler M (2017). High-resolution non-destructive three-dimensional imaging of integrated circuits. Nature.

[9] Holler M (2019). Three-dimensional imaging of integrated circuits with macro- to nanoscale zoom. Nat. Electron..

[10] Park JY, Kim Y, Lee S, Lim J (2020). Zernike phase-contrast full-field transmission x-ray nanotomography for 400 micrometre-sized samples. J. Synchrotron Rad..

[11] Guruswamy T, Gades L, Miceli A, Patel U, Quaranta O (2021). Beamline spectroscopy of integrated circuits with hard x-ray transition edge sensors at the Advanced Photon Source. IEEE Trans. on Appl. Supercond..

[12] Kalasová D (2019). Characterization of a laboratory-based x-ray computed nanotomography system for propagation-based method of phase contrast imaging. IEEE Transactions on Instrum. Meas..

[13] Flenner S (2020). Hard x-ray nano-holotomography with a Fresnel zone plate. Opt. Exp..

[14] Müller D (2021). Laboratory-based nano-computed tomography and examples of its application in the field of materials research. Crystals.

[15] Lutter F (2021). Combining x-ray nano tomography with focused ion beam serial section imaging-application of correlative tomography to integrated circuits. Nucl. Instrum. Methods Phys. Res. B Beam Interactions Mate. Atoms.

[16] Scharf J (2022). Bridging nano-and microscale x-ray tomography for battery research by leveraging artificial intelligence. Nat. Nanotechnol..

[17] Frigo SP, Levine ZH, Zaluzec NJ (2002). Submicron imaging of buried integrated circuit structures using scanning confocal electron microscopy. Appl. Phys. Lett..

[18] Ercius P, Weyland M, Muller DA, Gignac LM (2006). Three-dimensional imaging of nanovoids in copper interconnects using incoherent bright field tomography. Appl. Phys. Lett..

[19] Zhang, D. et al. Fast, full chip image stitching of nanoscale integrated circuits. Tech. Rep., SRI International Princeton United States (2019).

[20] Bender H (2019). Combined STEM-EDS tomography of nanowire structures. Semicond. Sci. Technol..

[21] Wang, S., Gelb, J., Lau, S. & Yun, W. Metrology of 3D IC with X-ray Microscopy and nano-scale X-ray CT. In *2009 IEEE International Interconnect Technology Conference*, 131–133 (IEEE, 2009).

[22] Kutukova K (2018). A laboratory x-ray microscopy study of cracks in on-chip interconnect stacks of integrated circuits. Appl. Phys. Lett..

[23] Silomon J, Gluch J, Clausner A, Paul J, Zschech E (2021). Crack identification and evaluation in BEoL stacks of two different samples utilizing acoustic emission testing and nano x-ray computed tomography. Microelectron. Reliab..

[24] Ullom JN, Bennett DA (2015). Review of superconducting transition-edge sensors for x-ray and gamma-ray spectroscopy. Supercond. Sci. Technol..

[25] Weichman PB, Lavely EM (2020). Fluorescent x-ray scan image quality prediction. J. Hardware Syst. Sec..

[26] Szypryt P (2021). Design of a 3000 pixel transition-edge sensor x-ray spectrometer for microcircuit tomography. IEEE Trans. Appl. Superconductivity.

[27] Nakamura, N. et al. A tabletop x-ray tomography instrument for nanometer-scale imaging: Integration of a scanning electron microscope with a transition-edge sensor spectrometer. *arXiv*. https://arxiv.org/abs/2212.10591 (2022).

[28] Moon, P. et al. A Cu interconnect process for the 130 nm process technology node. In *Advanced Metallization Conference (AMC)*, 39–41 (2001).

[29] Li B, Sullivan TD, Lee TC, Badami D (2004). Reliability challenges for copper interconnects. Microelectron. Reliab..

[30] Barrett, H. H. & Myers, K. J. *Foundations of Image Science* (John Wiley & Sons, 2013).

[31] Levine ZH, Blattner TJ, Peskin AP, Pintar AL (2019). Scatter corrections in x-ray computed tomography: a physics-based analysis. J. Res. Natl. Instit. Standards Technol..

[32] Visser, J. Tungsten CMP applications. Microelectronic Applications of Chemical Mechanical Planarization. 277 (2007).

[33] Li, Y. Why CMP? In Li, Y. (ed.) *Microelectronic Applications of Chemical Mechanical Planarization*, chap. 1, p.1 (Wiley, Hoboken, NJ, USA, 2008).

[34] Kyrieleis A, Titarenko V, Ibison M, Connolley T, Withers P (2011). Region-of-interest tomography using filtered backprojection: assessing the practical limits. J. Micro..

[35] Bouman C, Sauer K (1993). A generalized Gaussian image model for edge-preserving MAP estimation. IEEE Trans. Image Process..

[36] Sauer K, Bouman C (1993). A local update strategy for iterative reconstruction from projections. IEEE Trans. Signal Process..

[37] Sidky EY, Pan X (2008). Image reconstruction in circular cone-beam computed tomography by constrained, total-variation minimization. Phys. Med. Biol..

[38] Chityala, R. N., Hoffmann, K. R., Bednarek, D. R. & Rudin, S. Region of interest (ROI) computed tomography. In *Medical Imaging 2004: Phys. Med. Imaging*. **5368**, 53–4541 (International Society for Optics and Photonics, 2004). 10.1117/12.534568 PMC3033559 21297901

[39] Kalukin, A. R., Levine, Z. H., Frigo, S. P., McNulty, I. & Kuhn, M. Effects of feature orientation in tomographic reconstructions. In *X-Ray Microfocusing: Appl. Tech.* **3449**, 36–44 (SPIE, 1998).

[40] Kalukin, A. R. et al. Methods to remove distortion artifacts in scanned projections. In *Developments in X-Ray Tomography II*, vol. 3772, 237–245 (SPIE, 1999).

[41] Förster A, Brandstetter S, Schulze-Briese C (2019). Transforming x-ray detection with hybrid photon counting detectors. Philos. Trans. Royal Soc. A.

[42] Fowler JW (2016). The Practice of Pulse Processing. J. Low Temp. Phys..

[43] Szypryt P (2019). A transition-edge sensor-based x-ray spectrometer for the study of highly charged ions at the National Institute of Standards and Technology electron beam ion trap. Rev. Sci. Instrum..

[44] Szypryt, P. et al. A tabletop x-ray tomography instrument for nanometer-scale imaging: demonstration of the 1,000-element transition-edge sensor subarray. *IEEE Trans. Appl. Supercond., in press; early access* https://ieeexplore.ieee.org/document/10068315 (2023).

[45] Paganin, D. M. Coherent X-Ray Optics (Oxford University Press, Oxford, 2006).

[46] Levine ZH (2021). X-ray computed tomography using partially coherent Fresnel diffraction with application to an optical fiber. Opt. Express.

[47] Tang J, Nett BE, Chen G-H (2009). Performance comparison between total variation (TV)-based compressed sensing and statistical iterative reconstruction algorithms. Phys. Med. Biol..

[48] Berger, M. J. et al. XCOM: Photon Cross Sections Database, NIST Standard Reference Database 8 (XGAM), NBSIR 87-3597 (2010). Retrieved from 10.18434/T48G6X.

[49] Grodzins L (1983). Optimum energies for x-ray transmission tomography of small samples: Applications of synchrotron radiation to computerized tomography I. Nucl. Inst. and Meth. in Phys. Res..

[50] NEA (2019), PENELOPE 2018: A code system for Monte Carlo simulation of electron and photon transport: Workshop Proceedings, Barcelona, Spain, 28 January - 1 February 2019, OECD Publishing, Paris, 10.1787/32da5043-en.

[51] Guo Z (2022). Physics-assisted generative adversarial network for x-ray tomography. Opt. Express.

[52] Kremer JR, Mastronarde DN, McIntosh JR (1996). Computer visualization of three-dimensional image data using IMOD. J. Struct. Biol..

[53] Henke, B., Gullikson, E., & Davis, J. X-ray interactions: photoabsorption, scattering, transmission, and reflection at *E*=50-30000 eV, *Z*=1-92. At. Data Nuc. Data Tables 54, 181–342 (1993). Retrieved from https://henke.lbl.gov/optical_constants/filter2.html.

